# Manganese activates autophagy to alleviate endoplasmic reticulum stress–induced apoptosis via PERK pathway

**DOI:** 10.1111/jcmm.14732

**Published:** 2019-10-22

**Authors:** Chang Liu, Dong‐Ying Yan, Can Wang, Zhuo Ma, Yu Deng, Wei Liu, Bin Xu

**Affiliations:** ^1^ Department of Environmental Health School of Public Health China Medical University Shenyang China

**Keywords:** autophagy, endoplasmic reticulum stress, manganese, neurotoxicity, PERK signalling pathway

## Abstract

Overexposure to manganese (Mn) is neurotoxic. Our previous research has demonstrated that the interaction of endoplasmic reticulum (ER) stress and autophagy participates in the early stage of Mn‐mediated neurotoxicity in mouse. However, the mechanisms of ER stress signalling pathways in the initiation of autophagy remain confused. In the current study, we first validated that ER stress–mediated cell apoptosis is accompanied by autophagy in SH‐SY5Y cells. Then, we found that inhibiting ER stress with 4‐phenylbutyrate (4‐PBA) decreased ER stress–related protein expression and reduced cell apoptosis, whereas blocking autophagy with 3‐methyladenine (3‐MA) increased cell apoptosis. These data indicate that protective autophagy was activated to alleviate ER stress–mediated apoptosis. Knockdown of the protein kinase RNA‐like ER kinase (PERK) gene inhibited Mn‐induced autophagy and weakened the interaction between ATF4 and the LC3 promoter. Our results reveal a novel molecular mechanism in which ER stress may regulate autophagy via the PERK/eIF2α/ATF4 signalling pathway. Additionally, Mn may activate protective autophagy to alleviate ER stress–mediated apoptosis via the PERK/eIF2α/ATF4 signalling pathway in SH‐SY5Y cells.

## 1 INTRODUCTION

Manganese (Mn) is a metallic element which is vital during human development and is involved in several significant physiological processes that are required for various enzymatic reactions and neurological function. Whereas the major source of Mn absorption is dietary, occupational exposures to high dosage of inhaled Mn can produce toxic sequelae. Overexposure to Mn can result in neurotoxicity, as Mn easily crosses blood‐brain barrier and accumulates predominantly in the striatum, which results in a neurological disorder, known as manganism.^1^ The neurotoxicity of Mn was first associated with a neurodegenerative motor neuron disease caused by over‐accumulation of Mn in basal ganglia, which exhibited neurological symptoms similar to those of Parkinson's disease.^2^ Although numerous researches have studied Mn‐induced neurotoxicity, its mechanisms remain obscure. It has been demonstrated that Mn can produce reactive oxygen species (ROS), contribute to mitochondrial dysfunction, damage endoplasmic reticulum (ER) homeostasis, and promote protease activation and apoptotic cell death.^3^, ^4^ Mn can also initiate excitotoxic cell death by altering neurotransmitter levels. ER stress and ER stress–mediated apoptosis have been found to be participated in Mn‐induced neurotoxicity in vivo.^5^

Abnormal function of the ER can cause the unfolded protein response (UPR) to the cellular stress, which is originally a self‐defence mechanism that attempts to compensate for damage and thus promotes cell survival. The UPR is a complex cellular response that is transduced by three ER signalling pathways: PERK/eIF2α/ATF4, IRE‐1/Xbp‐1 and ATF6 to maintain ER homeostasis. Our previous study found that Mn could activate PERK and IRE1 signalling pathway, which contributed to the occurrence of apoptosis.^4^ ER stress–mediated cell apoptosis signalling is activated if the UPR fails to correct misfolded proteins in the ER.^6^ However, the mechanisms of ER stress–mediated cell apoptosis remain obscure, and there is far too little distinction regarding which specific effectors of death dominate in specific cellular environment. Furthermore, increasing researches have suggested a role of ER stress–mediated apoptosis in the physiopathology of manganism.^5^, ^7^

Recently, Mn has also been reported to activate protective autophagy in cells.^8^ As a regulatory response to protect against stress, autophagy recycles and degrades cellular components, organelles and proteins to maintain cell survival and homeostasis. However, the molecular mechanisms by which Mn‐induced autophagy are still not well clarified. Autophagy that is closely associated with cell apoptosis and promotes cell survival under stress conditions has been reported.^9^ Also, emerging evidence demonstrates that the ER provides membrane that is needed for the formation of autophagosomes and is critical for ER homeostasis.^10^ Nevertheless, there is little research detecting the effect of ER stress signalling pathways in the induction of autophagy. Furthermore, autophagy can selectively occur in certain conditions such as the disruption of ER homeostasis and can lead to the inhibition of apoptosis.^11^ Thus, selective autophagy may be beneficial to protect cells from excessive apoptosis.

Activation of the protein kinase RNA‐like ER kinase signalling (PERK) pathway plays a pivotal role in ER stress–mediated apoptosis and is almost simultaneous with the initiation of ER stress and is more sensitive than the inositol‐requiring enzyme 1 (IRE1) and activating transcription factor 6 (ATF6) signalling pathways.^4^, ^12^ Therefore, we hypothesized that the PERK/eIF2α/ATF4 signalling pathway could be involved in the induction of protective autophagy during early Mn exposure. The current study was designed to evaluate ER stress–mediated cell apoptosis and to explore the molecular mechanisms of the PERK/eIF2α/ATF4 signalling pathway in inducing protective autophagy in Mn‐treated SH‐SY5Y cells. This study has revealed that Mn can initiate protective autophagy via the PERK/eIF2α/ATF4 signalling pathway to alleviate ER stress–induced apoptosis.

## 2 MATERIALS AND METHODS

### 2.1 Chemicals and reagents

Manganese (II) chloride tetrahydrate (MnCl_2_.4H_2_O), 4‐phenylbutyric acid (4‐PBA), 3‐methyladenine (3‐MA) and monodansylcadaverine (MDC) were purchased from Sigma. Bafilomycin A1 (Baf‐A1) was obtained from MedChemExpress LLC. Annexin V‐FITC/PI detection kit was obtained from Life Technologies. PrimeScript^®^ RT Enzyme Mix I and SYBR^®^ Premix Ex TaqTM II kit were acquired from TaKaRa Biotech. Co. Ltd. Chromatin immunoprecipitation assay kit was purchased from Cell Signaling Technology, Inc. Ad‐mCherry‐GFP‐LC3B (adenovirus expressing mCherry‐GFP‐LC3B fusion protein) was obtained from Beyotime Biotechnology. The anti‐β‐actin, anti‐LC3B, anti‐beclin 1, anti‐p62, anti‐GRP78, anti‐PARP, anti‐cleaved PARP, anti‐CHOP, anti‐GADD34, anti‐total PERK and anti‐phospho‐PERK were acquired from Abcam Ltd. Anti‐total eIF2α, anti‐phospho‐eIF2α and anti‐ATF4 were obtained from Cell Signaling Technology. Horseradish peroxidase (HRP)–conjugated secondary antibody was from Abcam.

### 2.2 Cells and cell culture

The human neuroblastoma cell line (SH‐SY5Y, purchased from ATCC, CRL‐2266) was grown in DMEM with equal amount of F‐12 medium supplemented with 10% foetal bovine serum (FBS) and 1% penicillin‐streptomycin. The culture was maintained in humidified atmosphere at 37°C with 5% CO_2_.

### 2.3 Drug treatment

The experiment was divided into five parts; in the first part, SH‐SY5Y cells were exposed to 100 μmol/L Mn for different stages (6, 12, 24 hours) in serum‐free medium to evaluate the cytotoxicity of Mn. In the second and third parts, cells were exposed to Mn (0 and 100 μmol/L) for 24 hours and 4‐PBA (1 mmol/L, 2 mmol/L)^13^ or 3‐MA (5 μmol/L, 10 μmol/L)^14^ in serum‐free medium, and for 4‐PBA or 3‐MA experiments, cells were maintained in these media for 12 hours before Mn treatment. To block autophagic flux, cells were treated with 100 nmol/L bafilomycin A1 (Baf‐A1) alone or pretreatment for 2 hours before incubation with 100 μmol/L Mn for 24 hours.^15^, ^16^ In the fourth part, cultured cells, LV‐PERK shRNA and LV‐negative shRNA cells were, respectively, exposed to 0 and 100 μmol/L Mn for 24 hours in a serum‐free medium. In the fifth part, cells were exposed to Mn (0 and 100 μmol/L) and H_2_O_2_ (100 μmol/L), cells were maintained in H_2_O_2_ media for 12 hours before Mn treatment, and then, LV‐PERK shRNA was used into the H_2_O_2_ pretreatment cells.

### 2.4 Cytotoxicity assay

To analyse cell cytotoxicity, approximately 1 × 10^4^ cells/well were cultured and grown onto each 96‐well plate for 24 hours. Then, the medium was replaced with 100 μmol/L Mn for different stages (6, 12, 24 hours). Cell viability of neuronal cell was quantitatively detected with CCK‐8 assay. The CCK‐8 reagent (Sigma) was added into wells 2 hours before the completion of the treatment period, and cell viability (%) was calculated using the following formula: (treated groups OD values/ control group OD values)×100%.

LDH is released from cells after injury and is thus used to evaluate the integrity of cell membrane. After Mn treatment, LDH release was detected according to the manufacturer's methods. The absorbance values were determined by using the microplate reader at 490 nm, and the absorbance results of test wells were shown as percentage of the control well.

### 2.5 Apoptosis assay

To analyse cell apoptosis, approximately 1 × 10^6^ cells were collected and mixed in 500 μL of binding buffer containing 5 μL AnnexinV‐FITC and 5 μL PI after rinsed twice with cold 1 × PBS. The mixture was incubated at room temperature for 20 minutes in dark. Cell apoptosis was detected by an FCM (Becton‐Dickinson) and Cell Quest software. The percentage of single positive populations (FITC＋/PI－) in quadrant Q4 was regarded to exhibit an early apoptosis rate.

### 2.6 Detection of autophagic vacuoles with MDC

In order to detect the autophagic vacuole formation, monodansylcadaverine (MDC) (50 μmol/L) was added into cells and incubated at 37°C for 45 minutes in dark. After staining with MDC, before analysed with flow cytometry (FCM), the cells were rinsed twice with 1 × PBS. The results are shown as the mean fluorescence percentage (%) of control.

### 2.7 Detection of intracellular ROS by flow cytometry

In order to detect intracellular ROS production, DCFH‐DA (10 μmol/L) was added into cells and incubated at 37°C for 20 minutes in dark. Then, the cells were collected and resuspended in 500 μL of PBS buffer. Then, intracellular ROS levels were performed by FCM, and all determinations were performed at least four times.

### 2.8 Western blotting

Total proteins from SH‐SY5Y cells were extracted according to instruction procedures. SDS‐PAGE was used to separate equal amounts of total proteins (20 μg) that were transferred to PVDF (polyvinylidene fluoride) membranes (Millipore, Ternicula, CA). Then, the samples were blocked with 5% skimmed milk for 2 hours and incubated overnight with appropriate primary antibodies at 4°C. After incubation with matched secondary antibody at temperature for 2 hours, the target protein signal was measured using enhanced chemiluminescence (ECL) and analysed with an image software (FluorChem v2.0). The specific protein band expression was quantified and normalized to β‐actin. Experiments were repeated at least four times.

### 2.9 Reverse transcription quantitative PCR (RT‐qPCR)

Total RNA was extracted from SH‐SY5Y cells with TRIzol reagent (Invitrogen), and cDNA was synthesis by performing with Prime‐Script RT reagent kit (TaKaRa Bio Inc). The sequences of primers were as follows: human β‐actin (forward: 5'‐CTA CCT CAT GAA GAT CCT CAC CGA‐3', reverse: 5'‐TTC TCC TTA ATG TCA CGC ACG ATT‐3'); and human LC3 (forward: 5'‐TAC GGA AAG CAG CAG TGT‐3', reverse: 5'‐GAA GGC AGA AGG GAG TGT‐3').^17^ RT‐qPCR was performed with SYBR Green Premix and measured by ABI 7500 RT‐qPCR System (Applied Biosystems). Each sample was detected in quadruplicate. The relative quantification of gene expression was measured by comparative 2^‐ΔΔCt^ cycle threshold method after normalizing to β‐actin.

### 2.10 Lentiviral vector construction and transfection assay

In order to knockdown human PERK (sense: 5'‐GCG GCA GGU CAU UAG UAA U‐3'), the pGCSIL‐GFP‐PERK shRNA and PERK‐RNA interference (RNAi) lentiviral vectors were constructed, and using the Lentivector Expression System (Shanghai GeneChem Co, Ltd.) to package the recombinant virus as described previously.^14^ The negative control used was scrambled (Scr) shRNA. Cultured cells at 5‐7 days were cotransfected for 72 hours with the recombinant lentivirus, until more than 90% green fluorescent protein in LV‐PERK shRNA cells (transfected with PERK shRNA) and LV‐negative shRNA cells (transfected with Scr shRNA) was observed under a fluorescence microscope. Verification of gene silencing efficiency was conducted with Western blotting. After confirmation of optimal transfection and silencing efficiency, the following assays were performed.

### 2.11 Chromatin immunoprecipitation (ChIP) assay

SH‐SY5Y cells were cultured at approximately 8 × 10^6^ cells/15 cm culture dish and grown for 24 hours. The protein complexes were cross‐linked to their bound DNA by adding final concentration of 1% formaldehyde at room temperature for 10 minutes, and then, glycine was employed to quench the reaction. The cross‐linked chromatin was digested by adding 0.5 μL micrococcal nuclease for 20 minutes at 37°C to acquire 150‐900 bp DNA fragments. The liquid supernatant was divided into equal amount after centrifugation (10 000rpm, 4°C, 10 minutes). Afterwards, each aliquot was incubated with anti‐ATF4 anti‐rabbit IgG at 4°C overnight with gentle rotation. The antibody‐protein‐DNA complexes was isolate by using ChIP‐grade protein G magnetic beads to for 2 hours at 4°C with rotation and reversed by heating for 30 minutes at 65°C to release the DNA fragments, then purified using the spin columns. The reacquired DNA was performed with the SYBR Green Assay and analysed using the ABI 7500 RT‐qPCR System. The ChIP‐PCR LC3B promoter was forward: 5'‐GCA GCA CCA CCA AGT CTC TC‐3' and reverse: 5'‐ACT CTT GAG GGA GGG GTC AG‐3'.^18^ With this method, each immunoprecipitation signals were expressed as a per cent of the total chromatin.

### 2.12 Ad‐mCherry‐GFP‐LC3B transfection assay

To analyse autophagic flux, cells were grown onto 48‐well cell culture plates and transfected at 20%–30% confluence. SH‐SY5Y cells were infected with Ad‐mCherry‐GFP‐LC3B (Cat No: C3011) adenovirus at a MOI of 80. After 48 hours, the following treatment was performed on cells. The expression of autophagy flux was visualized with a confocal laser scanning microscopy (FV1000S‐IX81, Olympus). Autophagy flux was examined by counting the change of fluorescence signal (red and yellow signals).

### 2.13 Statistical analysis

Data are expressed as mean ± SD of four independent experiments. All statistical analyses were performed with SPSS 18.0, and differences were determined by one‐way ANOVA followed by a Student‐Newman‐Keuls test for multiple comparisons. *P* < .05 or *P* < .01 indicated statistically significant differences.

## 3 RESULTS

### 3.1 Manganese n induces cell injury and apoptosis in SH‐SY5Y cells

**Figure 1:**
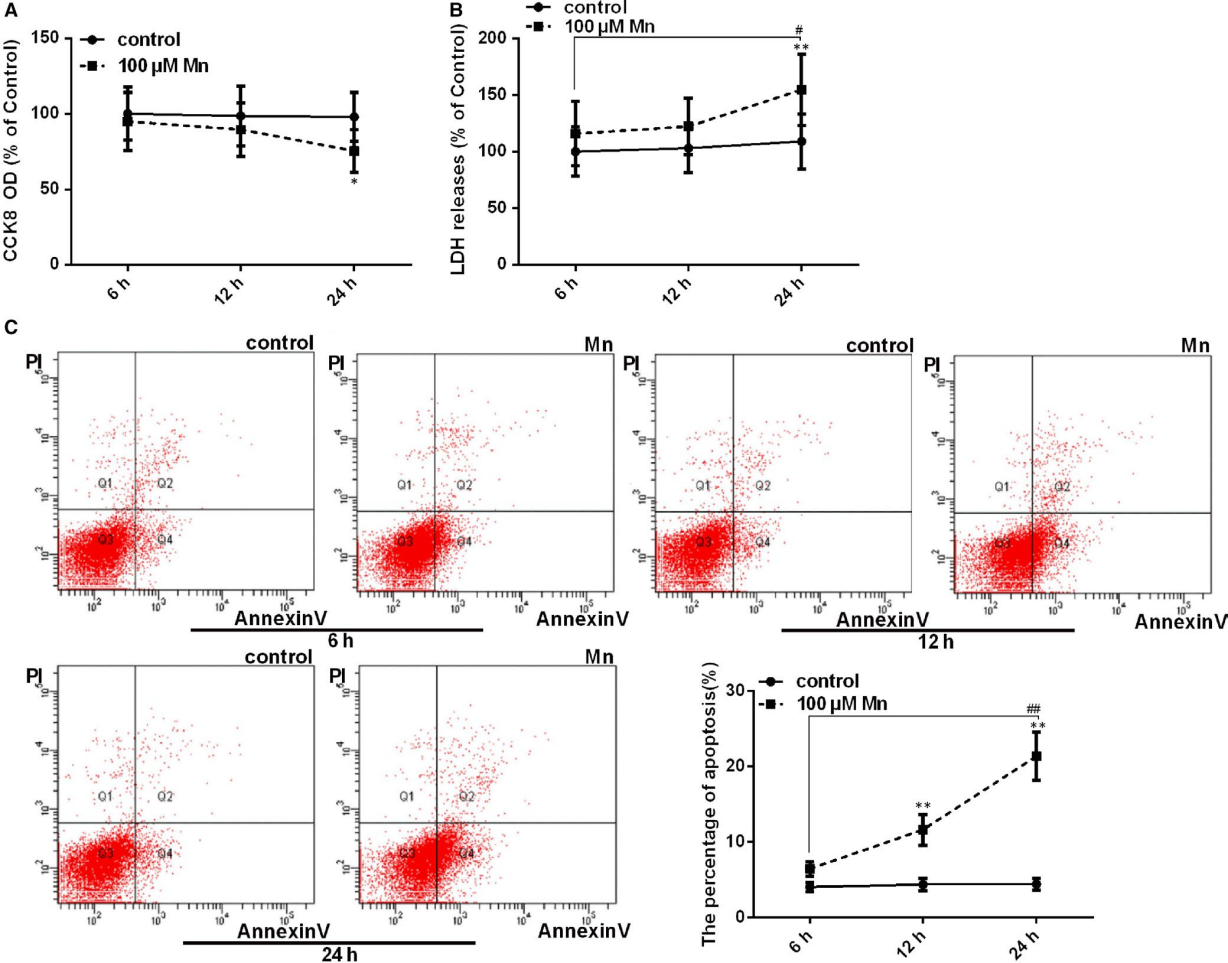
Manganese induces cell injury and apoptotic cell death in SH‐SY5Y cells. After treatment with Mn for 6, 12 and 24 h. (A) The CCK‐8 assay was used to measure cell viability by using the microplate reader at 450 nm shown in a line graph (B) The LDH release measured using the microplate reader at 490 nm is shown in a line graph. (C) The apoptosis percentage of cells (Annexin V+/PI−, Q4) was regarded as the early apoptosis rate, and the effect on Mn‐induced neural apoptosis is shown in a line graph. *P < .05, **P < .01, compared to controls at the same point, ^#^ *P* < .05, ^##^ *P* < .01, compared to the cells treated for 6 h

To evaluate the neurocytotoxicity of Mn, we detected the cell viability, LDH release, and percentage of early cell apoptosis. Mn was applied at a concentration of 100 μmol/L for different time periods (6, 12, 24 hours). Following treatment, the cell viability was significantly decreased in time dependence (*P < .*05, Figure 1A), and LDH release was significantly increased in time dependence (*P* < .01, Figure 1B). These results demonstrate that Mn can induce cell injury in SH‐SY5Y cells. The percentage of cells apoptosis was also measured with FCM with double staining of AnnexinV‐FITC and PI. The percentage of apoptotic cells also increased (*P* < .01, Figure 1C) up to 4.82‐fold after Mn treatment for 24 hours. Altogether, these results imply that Mn can induce apoptotic cell death.

### 3.2 Endoplasmic reticulum stress–mediated apoptosis is involved in Mn‐induced cell death

**Figure 2:**
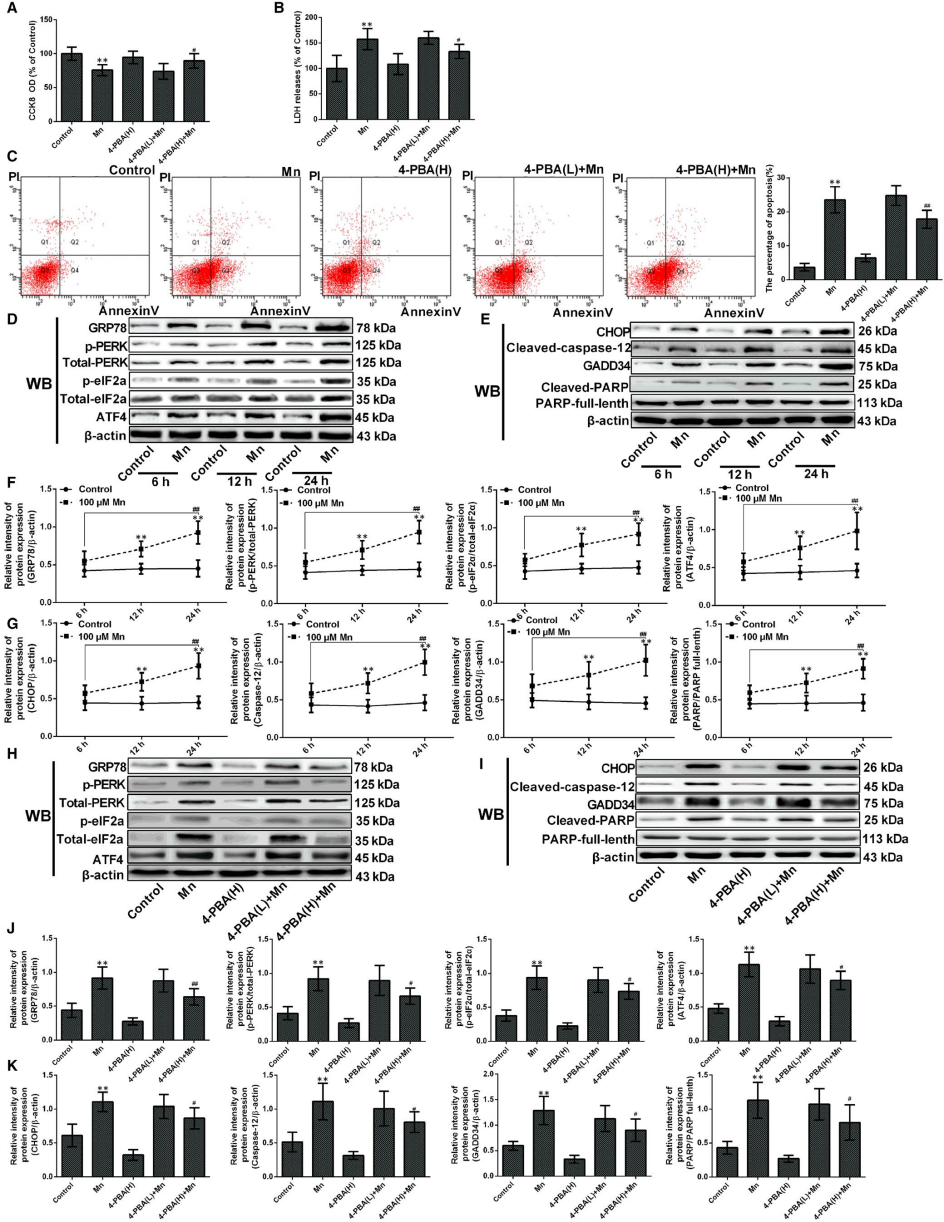
Endoplasmic reticulum stress–mediated apoptosis was involved in Mn‐induced cell death. After treatment with Mn and pretreated with 4‐PBA. (A) The CCK‐8 assay was used to measured cell viability by using the microplate reader at 450 nm shown in a bar graph. (B) The LDH release measured using the microplate reader at 490 nm is shown in a bar graph. (C) The percentage of single positive populations (FITC +/PI –) in quadrant Q4 was regarded as the early apoptosis rate. The early apoptosis in neuron cell by flow cytometry (FCM) is shown in a bar graph. (D) (E) The bands of GRP78, phospho‐PERK, phospho‐eIF2α, ATF4, CHOP, Caspase‐12, PARP, GADD34 and β‐actin expression levels measured by Western blotting assay after treatment with Mn for 6, 12 and 24 h. (F) The relative intensity of GRP78, PERK, phospho‐PERK, eIF2α and phospho‐eIF2α and expression of ATF4 after treatment with Mn for 6 h, 12 h and 24 h are shown in a line graph. (G) The protein CHOP, Caspase‐12, PARP and GADD34 expressions after treatment with Mn for 6, 12 and 24 h are shown in a line graph. (H, I) The bands of GRP78, phospho‐PERK, phospho‐eIF2α, ATF4, CHOP, Caspase‐12, PARP, GADD34 and β‐actin expression levels measured by Western blotting assay after treatment with Mn pretreated with high‐dose 4‐PBA. (J) The relative intensity of GRP78, PERK, phospho‐PERK, eIF2α and phospho‐eIF2α and expression of ATF4 are shown in a bar graph after treatment with Mn and pretreated with high‐dose 4‐PBA. (K) The protein CHOP, Caspase‐12, PARP and GADD34 expressions after treatment with Mn and pretreated with high‐dose 4‐PBA are shown in a bar graph. *P < .05, **P < .01, compared to the controls; ^#^ *P* < .05, ^##^ *P* < .01, compared to the cells treated for 6 h or 100 μmol/L Mn‐treated group; H, high dose, L, low dose

To verify that ER stress was contributing to Mn‐induced cell death, SH‐SY5Y cells were exposed to 100 μmol/L Mn and/or 2 mmol/L 4‐phenylbutyric acid (4‐PBA). 4‐PBA is a specific inhibitor of ER stress and was employed as a negative control. The cytotoxicity of 4‐PBA was estimated with CCK‐8 and LDH release measurements. Interestingly, pretreatment with 4‐PBA reduced Mn‐induced cytotoxicity (*P* < .05, Figure 2A). Cells pretreated with 4‐PBA exhibited significant reduction on LDH release compared with cells only exposed to Mn (*P* < .05, Figure 2B). There was no distinct neurotoxicity in cells exposed to 2 mmol/L 4‐PBA alone compared with the control. These results suggest that 4‐PBA pretreatment can reduce Mn‐induced cell damage.

The percentage of apoptosis was significantly decreased after pretreatment with high‐dose 4‐PBA compared with only Mn treatment (24.31%, *P* < .01, Figure 2C), which indicates that ER stress contributes to Mn‐mediated cell death. To elucidate whether Mn‐induced cell apoptosis was related to ER stress, proteins involved in ER stress signal pathways were also evaluated. After Mn incubation for 6, 12, and 24 hours, the expression of GRP78, phospho‐PERK, phospho‐eIF2α and ATF4 was significantly increased (Figure 2D and F, *P* < .01), indicating that Mn exposure can initiate ER stress. Also, ER stress can activate apoptotic mediators, including the transcription factor C/EBP homologous protein (CHOP) and Caspase‐12, and our findings indicate that the expression of CHOP and cleaved Caspase‐12 significantly increased after Mn treatment for 24 hours. (2.02‐fold and 2.15‐fold, *P* < .01, Figure 2E and G). The protein phosphatase 1 regulatory subunit 15A, or MyD116 (GADD34), and cleaved poly (ADP‐ribose) polymerase (PARP) are mediators of apoptosis, and their expression also increased significantly (2.24‐fold and 1.97‐fold, *P* < .01, Figure 2E and G). To further confirm that ER stress can induce cell death, we examined the GRP78, phospho‐PERK, phospho‐eIF2α, ATF4, CHOP, GADD34, cleaved Caspase‐12 and cleaved‐PARP protein expression in 4‐PBA‐pretreated cells and found that the expression was significantly decreased compared with Mn‐treated cells (*P* < .05, Figure 2H‐K).

### 3.3 Protective autophagy is activated to alleviate Mn‐induced cell injury

**Figure 3:**
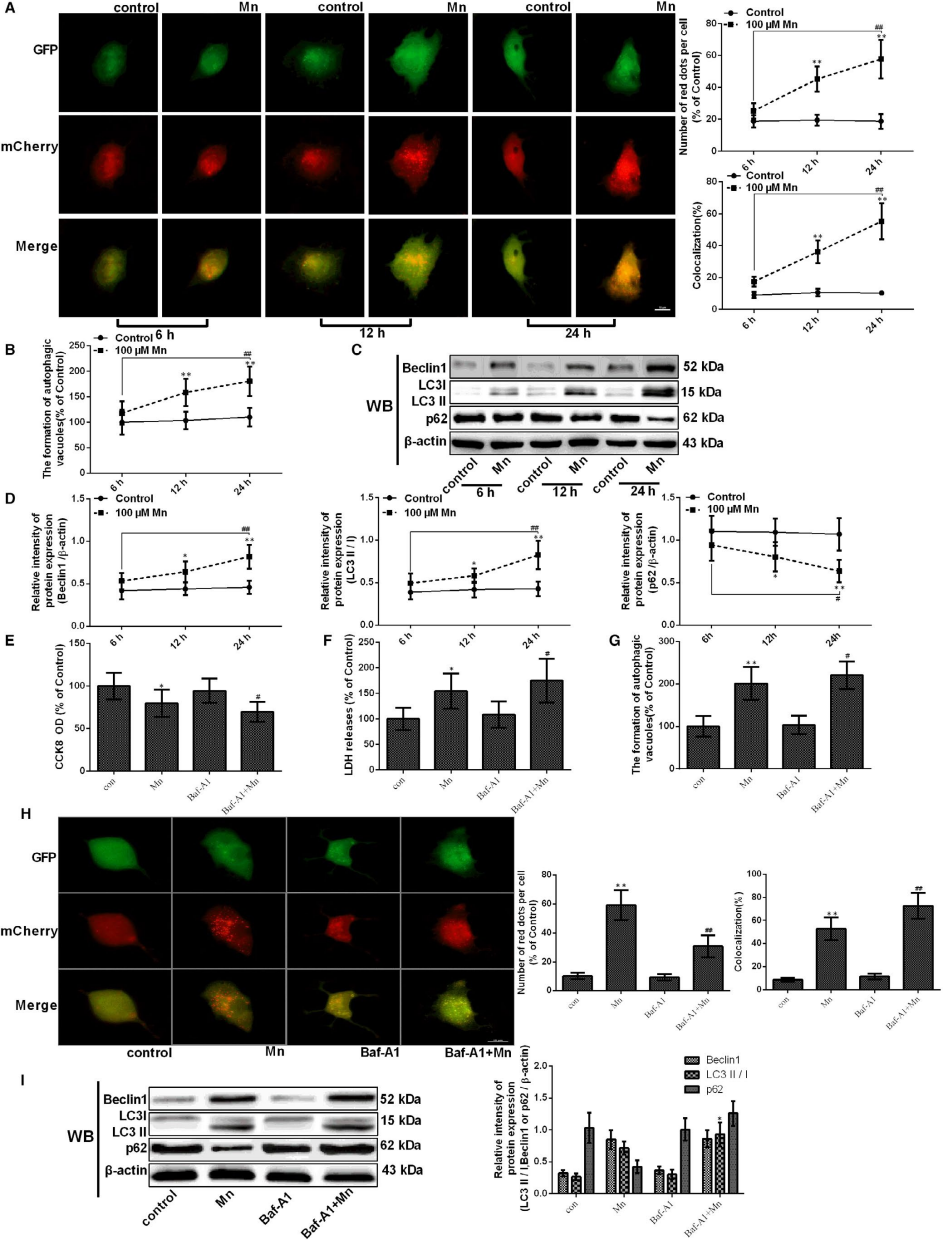
Manganese induces the activation of autophagy. After treatment with Mn for 6, 12 and 24 h. (A) The fluorescence signal change in normal and treatment cells after transfection with ad‐mCherry‐GFP‐LC3B adenovirus. (B) The measurement of autophagic vacuoles after staining with MDC detected by FCM is shown in a line graph. (C‐D) The bands of beclin‐1, LC3, p62 and β‐actin expression levels measured by using Western blotting in SH‐SY5Y cells, and the protein beclin‐1, LC3 and p62 expressions are shown in a line graph. (E) The CCK‐8 assay was used to measured cell viability by using the microplate reader at 450 nm shown in a bar graph. (F) The LDH release measured using the microplate reader at 490 nm is shown in a bar graph. (G) The measurement of autophagic vacuoles after staining with MDC detected by FCM is shown in a bar graph. (H) The fluorescence signal change in normal and treatment cells after transfection with ad‐mCherry‐GFP‐LC3B adenovirus. (I) The bands of beclin‐1, LC3, p62 and β‐actin expression levels measured by using Western blotting in SH‐SY5Y cells, and the protein beclin‐1, LC3 and p62 expressions are shown in a bar graph.*P < .05, **P < .01, compared to controls at the same point; ^#^ *P* < .05, ^##^ *P* < .01, compared to the cells treated for 6 h

In this study, ad‐mCherry‐GFP‐LC3B adenoviruses were transfected into SH‐SY5Y cells, which enables autophagy observation, as green fluorescence is quenched in acidic lysosomes. Thus, an increase in both red signal (mCherry fluorescence) and yellow signal (merged by GFP and mCherry fluorescence) indicates autophagy activation, while an increase in only yellow signal or the high colocalization of mCherry and GFP‐LC3 indicates the early formation of autophagosomes or the inhibition of autophagy degradation process. After Mn treatment, the red signal and the colocalization of mCherry and GFP‐LC3 were significantly increased in time dependence (*P* < .01, Figure 3A). MDC, a specific fluorescent labelling of autophagy flux for acidic compartments and lysosomes, has been applied to visualize the steps involved in autophagosome formation.^19^ MDC staining and flow cytometry indicated that the formation of autophagic vacuoles also increased significantly in time dependence (1.64‐fold, *P* < .01, Figure 3B). Western blotting revealed that beclin 1 expression and the ratio LC3II/LC3I increased significantly in a time‐dependent manner (1.79‐fold, *P* < .05 and 1.92‐fold*, P* < .01). However, p62 expression was significantly decreased after Mn treatment in time dependence (40.59%, *P* < .05, Figure 3C‐D).

To verify that Mn indeed enhanced autophagy induction, we further employed Baf‐A1, a selective inhibitor, which blocks the fusion of autophagosomes with lysosomes. To estimate Baf‐A1 cytotoxicity, we used CCK‐8 assay and LDH release to measure the viability of cell and membrane integrity. As the results showed, pretreatment with Baf‐A1 increased Mn‐induced cytotoxicity (*P* < .05, Figure 3E) and increased LDH release compared with cells only treated with Mn (*P* < .05, Figure 3F). pretreatment with Baf‐A1 decreased autophagy flux but increased the formation of autophagic vacuoles compared with only Mn treatment (*P* < .05 and *P < *.01, Figure 3G‐H). After Baf‐A1 pretreatment, the ratio of LC3II/LC3I was significantly increased (*P* < .05, Figure 3I), but not influences the expression of p62. These data indicate that Mn indeed enhanced autophagy induction.

**Figure 4:**
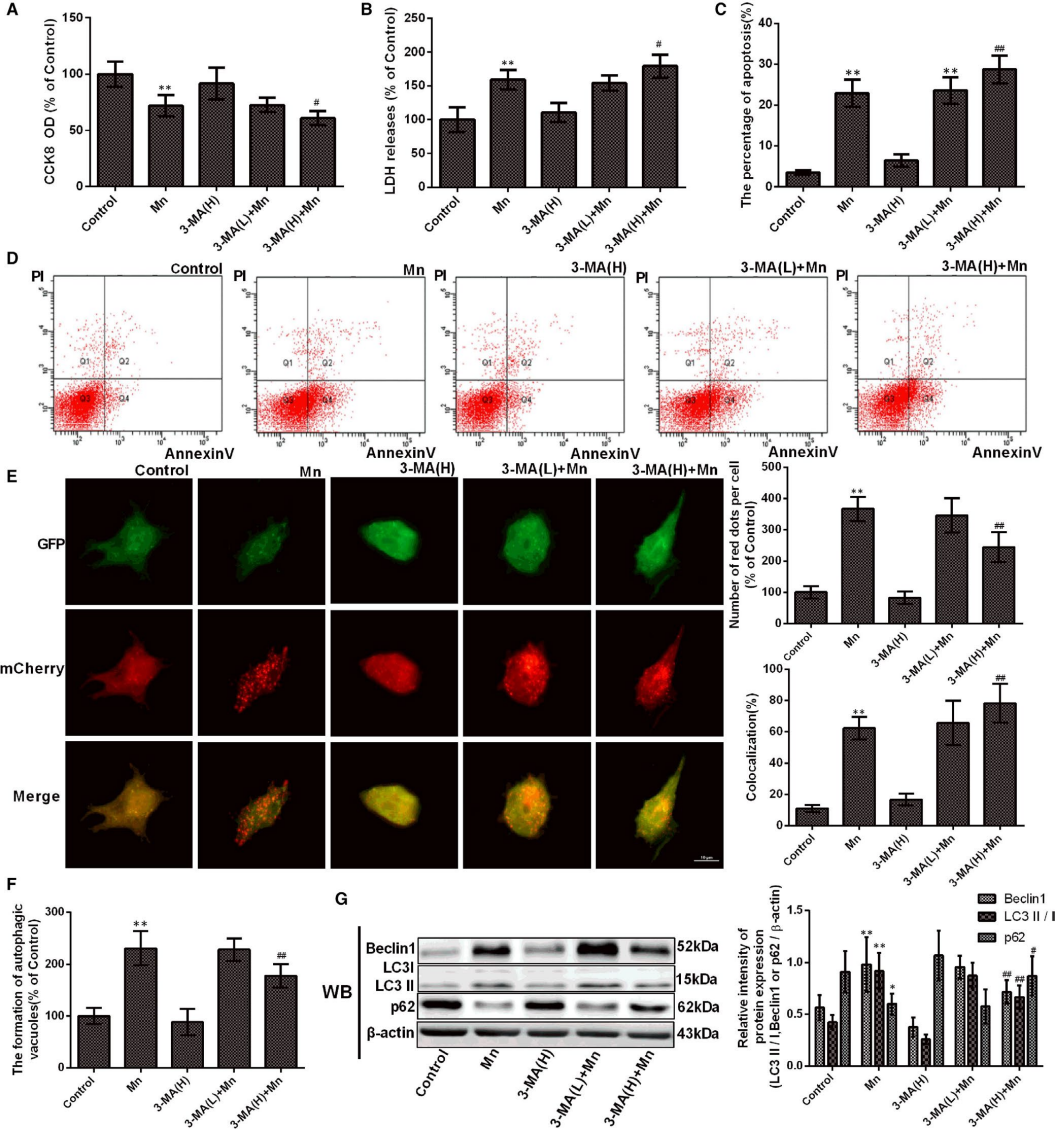
Protective autophagy was activated to alleviate Mn‐induced cell injury. After cells were treated with Mn and pretreated with 3‐MA. (A) The CCK‐8 assay was used to measure cell viability by using the microplate reader at 450 nm shown in a bar graph. (B) The LDH release measured using the microplate reader at 490 nm is shown in a bar graph. (C) The early apoptosis in neuron cell by flow cytometry (FCM) is shown in a bar graph. (D) The percentage of single positive populations (FITC +/PI –) in quadrant Q4 was regarded as the early apoptosis rate. (E) The fluorescence signal change in normal and treatment cells after transfection with ad‐mCherry‐GFP‐LC3B adenovirus. (F) The measurement of autophagic vacuoles after staining with MDC detected by FCM is shown in a bar graph. (G) The bands of beclin‐1, LC3, p62 and β‐actin expression levels measured by using Western blotting assay in SH‐SY5Y cells and the protein beclin‐1, LC3 and p62 after Western blotting experiments expressions are shown in a bar graph.*P < .05, **P < .01, compared to controls; ^#^ *P* < .05, ^##^ *P* < 01, compared to the 100μM Mn‐treated group; H, high dose, L, low dose

To further evaluate whether Mn‐induced autophagy could alleviate ER stress–induced cell apoptosis, 3‐methyladenine (3‐MA), a specific inhibitor of autophagosome formation, was used. To estimate 3‐MA cytotoxicity, we used CCK‐8 assay and LDH release to measure the viability of cell and membrane integrity. Pretreatment with 3‐MA increased Mn‐induced cytotoxicity (*P* < .05, Figure 4A) and increased LDH release compared with cells only treated with Mn (*P* < .05, Figure 4B). Treatment with only 3‐MA exhibited no effect on cells compared with the control. The percentage of apoptosis increased after 3‐MA pretreatment compared with only Mn‐treated cells (1.25‐fold, *P < *.01, Figure 4C‐D). High‐dose 3‐MA decreased autophagy flux and the formation of autophagic vacuoles compared with only Mn treatment (22.94%, *P < *.01, Figure 4E‐F). After high‐dose 3‐MA pretreatment, beclin1 expression and LC3II/LC3I significantly decreased (26.91% and 27.46%, *P* < .05), whereas p62 expression increased compared with Mn treatment (1.45‐fold, *P* < .05, Figure 4G). These data indicate that protective autophagy was activated to alleviate Mn‐induced cell injury.

### 3.4 Protein kinase RNA‐like ER kinase gene knockdown causes SH‐SY5Y cells to be susceptible to Mn

**Figure 5:**
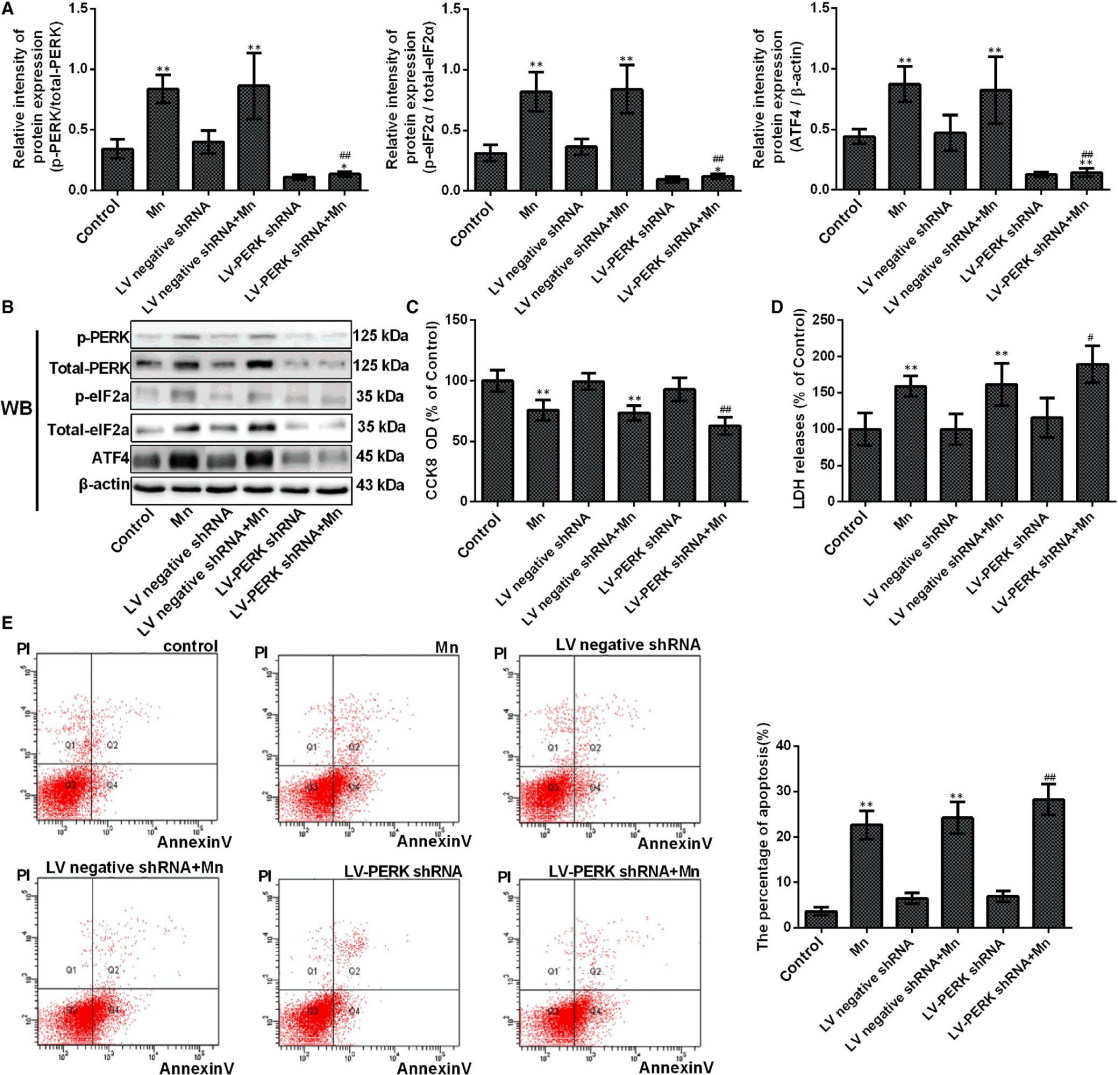
Protein kinase RNA‐like ER kinase gene knockdown causes SH‐SY5Y cells to be susceptible to Mn. After cells were transfected with PERK shRNA and treated with Mn. (A) The relative ratio of PERK, phospho‐PERK, eIF2α and phospho‐eIF2α and expression of ATF4 are shown in a bar graph. (B) The bands of PERK, phospho‐PERK, eIF2α, phospho‐eIF2α, ATF4 and β‐actin expression levels measured by using Western blotting assay in SH‐SY5Y cells. (C) The CCK‐8 assay was used to measure cell viability by using the microplate reader at 450 nm shown in a bar graph. (D) The LDH release measured using the microplate reader at 490 nm is shown in a bar graph. (E) The percentage of single positive populations (FITC +/PI –) in quadrant Q4 was regarded as the early apoptosis rate, and the early apoptosis in neuron cell by flow cytometry (FCM) is shown in a bar graph. *P < .05, **P < .01, compared to controls; ^#^ *P* < .05, ^##^ *P* < .01, compared to the 100 μmol/L Mn‐treated group

To evaluate whether the PERK signalling pathway is participated in Mn‐induced cell apoptosis, cells were transfected with the LV‐PERK shRNA. To detect the efficiency of knockdown, the PERK signalling pathway protein expression was examined in normal control cells, LV‐PERK shRNA transfected cells and LV‐negative shRNA transfected cells. Western blotting results showed that the protein levels of PERK, Phospho‐eIF2α and ATF4 in LV‐PERK shRNA cells were significantly weaker than the levels in the normal control and LV‐negative shRNA cultured cells (*P* < .01, Figure 5A‐B), and no distinctions were observed between the normal control and LV‐negative shRNA cultured cells. Mn‐treated LV‐PERK shRNA cell viability had a significant decline compared with the Mn‐treated normal cells; moreover, Mn‐treated LV‐negative shRNA cell viability had a significant decrease compared with untreated normal cells (*P* < .01, Figure 5C). LDH release in Mn‐treated LV‐PERK shRNA cells was distinctly increased compared with the Mn‐treated normal cells (*P* < .05, Figure 5D). Mn‐treated LV‐negative shRNA cells also exhibited a significant increase in LDH release compared with untreated normal cells. The percentage of apoptosis was increased in Mn‐treated LV‐PERK shRNA cells compared with only Mn‐treated cells (1.24‐fold, *P* <.01, Figure 4E). There were no obviously distinctions in CCK‐8, LDH release and apoptosis between LV‐PERK shRNA, LV‐negative shRNA and untreated normal cells. These results indicate that PERK gene knockdown may influence the activation of the PERK signalling pathway, thus causing SH‐SY5Y cells to be susceptible to Mn.

### 3.5 Protein kinase RNA‐like ER kinase signalling pathway participates in the initiation of protective autophagy

**Figure 6:**
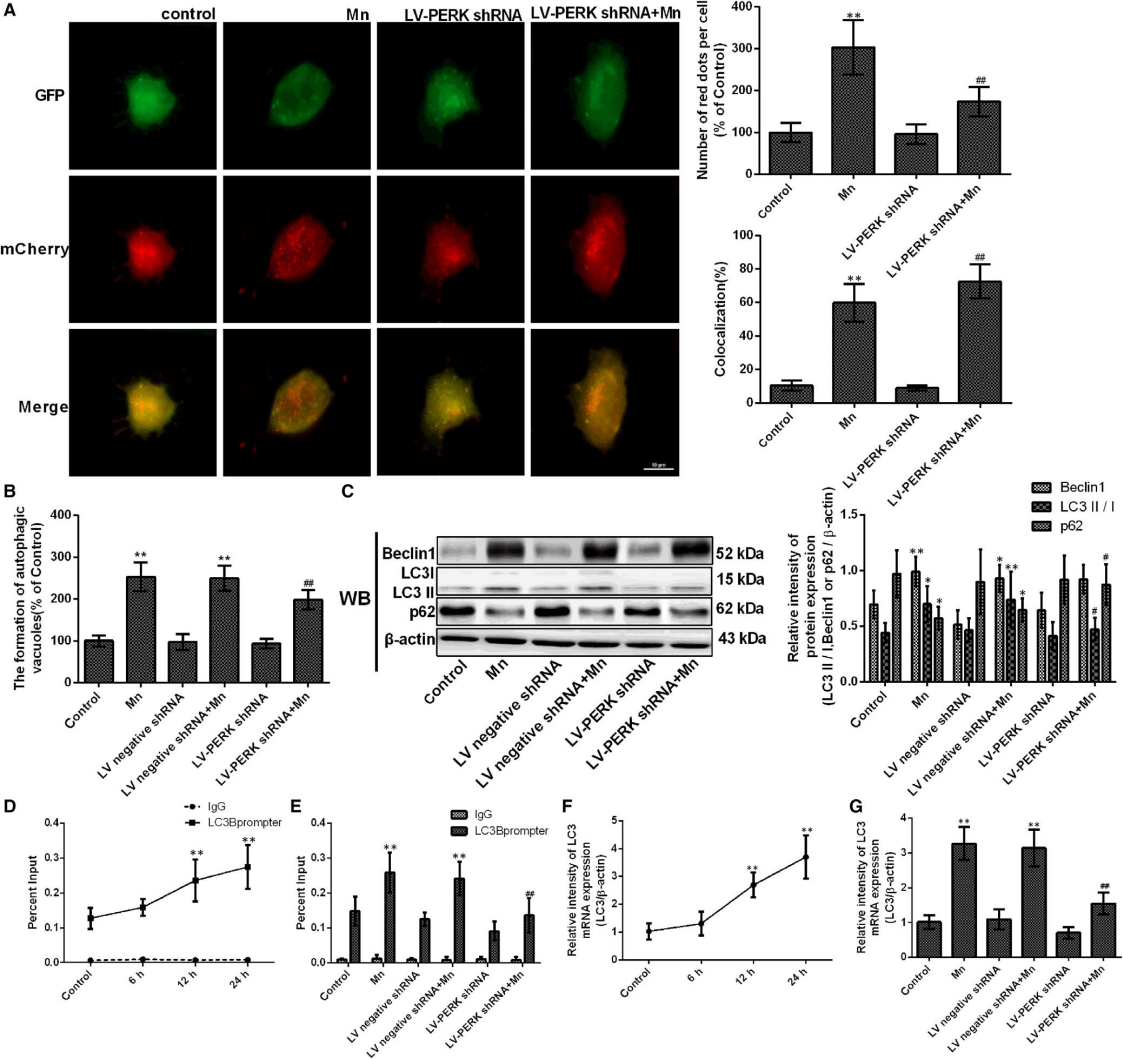
Protein kinase RNA‐like ER kinase signalling pathway participates in the initiation of protective autophagy. After cells were transfected with LV‐PERK shRNA and treated with Mn. (A) The fluorescence signal change in normal and treatment cells after transfection with ad‐mCherry‐GFP‐LC3B adenovirus. (B) The measurement of autophagic vacuoles after staining with MDC detected by FCM is shown in a bar graph. (C) The bands of beclin‐1, LC3, p62 and β‐actin expression levels measured by using Western blotting assay in SH‐SY5Y cells, and the protein beclin‐1, LC3 and p62 expressions are shown in a bar graph. (D) SH‐SY5Y cells were treatment with 100 μmol/L Mn for 6, 12 and 24 h, and the binding of ATF4 to the LC3 promoter by quantitative ChIP analysis is shown in line graph. (E) After cells were transfected with PERK shRNA and treated with Mn, the binding of ATF4 to the LC3 promoter by quantitative ChIP analysis is shown in a bar graph. (F) SH‐SY5Y cells were treated with 100 μmol/L Mn for 6, 12 and 24 h, and the expression levels of LC3 mRNA measured by RT‐qPCR assay are shown in a line graph. (G) The expression levels of LC3 mRNA measured by RT‐qPCR assay are shown in a bar graph. *P < .05, **P <.01, compared to controls; ^#^ *P* < .05, ^##^ *P* < .01, compared to the 100 μmol/L Mn‐treated group

To further analyse whether the PERK signalling pathway is essential to the activation of autophagy in Mn‐treated cells, we measured autophagy flux and the formation of autophagic vacuoles. Mn‐treated LV‐PERK shRNA cells exhibited a significant decrease in autophagy flux and formation of autophagic vacuoles compared with Mn‐treated normal cells (56.88%, *P* < .01, Figure 6A‐B). The ratio of LC3II/LC3I protein expression was decreased in Mn‐treated LV‐PERK shRNA cells compared with Mn‐treated normal cells (32.32%, *P* < .05). However, the p62 expression was significantly increased in Mn‐treated LV‐PERK shRNA cells compared with Mn‐treated normal cells (1.53‐fold, *P* < .05, Figure 6C).

LC3 is a key signalling molecule for autophagy initiation. To validate whether the expression of the LC3 gene is related to the PERK signalling pathway, we detected binding of ATF4 protein to the LC3 promoter in SH‐SY5Y cells using ChIP assays. Binding of ATF4 to the LC3 promoter and the mRNA expression of LC3 were highly enhanced in a time‐dependent manner following Mn treatment (2.15‐fold, *P* < .01, Figure 6D and F). However, binding of ATF4 to the LC3 promoter and the mRNA expression of LC3 were significantly weakened in Mn‐treated LV‐PERK shRNA cells compared with Mn‐treated normal cells (47.40% and 52.67%, respectively, *P* < .01, Figure 6E and G). This suggests that LC3 gene expression requires activation of the PERK signalling pathway and that ATF4 is responsible for LC3 transcription and expression. These findings indicate that Mn initiates the transformation from ER stress–induced apoptosis to autophagy as a protective response in SH‐SY5Y cells via the PERK signalling pathway.

### 3.6 The interaction of ATF4 to the LC3 promoter is directly regulated by the PERK signalling pathway

**Figure 7:**
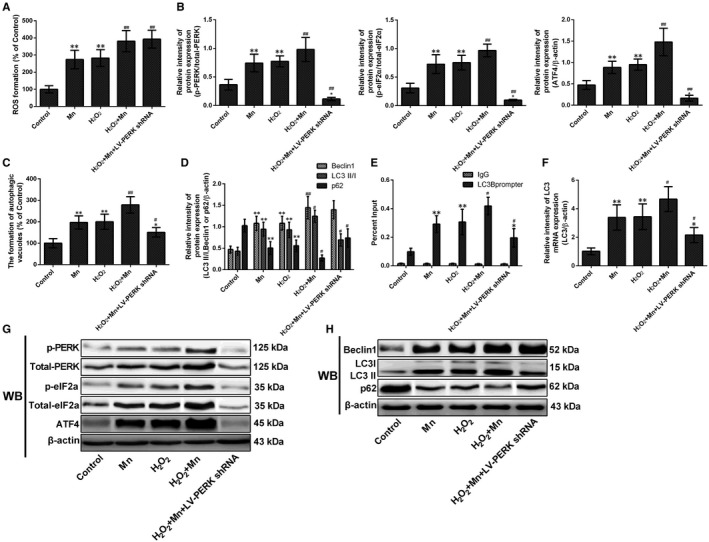
The interaction of ATF4 to the LC3 promoter is directly regulated by the PERK signalling pathway. After treatment with Mn, H_2_O_2_ and LV‐PERK shRNA. (A) The measurement of ROS after staining with DCFH‐DA detected by FCM was shown in a bar graph. (B) The relative intensity of PERK, phospho‐PERK, eIF2α and phospho‐eIF2α and expression of ATF4 are shown in a bar graph. (C) The measurement of autophagic vacuoles after staining with MDC detected by FCM is shown in a bar graph. (D) The protein beclin‐1, LC3 and p62 expressions are shown in a bar graph. (E) The binding of ATF4 to the LC3 promoter by quantitative ChIP analysis shown in bar graph. (F) The expression levels of LC3 mRNA measured by RT‐qPCR assay are shown in a bar graph. (G) The bands of PERK, phospho‐PERK, eIF2α, phospho‐eIF2α, ATF4 and β‐actin expression levels measured by using Western blotting assay in SH‐SY5Y cells. (H) The bands of beclin‐1, LC3, p62 and β‐actin expression levels measured by using Western blotting assay in SH‐SY5Y cells. *P < .05, **P < .01, compared to controls; ^#^ *P* < .05, ^##^ *P* < .01, compared to the 100 μmol/L Mn‐treated group

To evaluate the role of ROS on Mn‐induced autophagy activation, H_2_O_2_, a specific stimulator of ROS, was employed as a positive control. As the results showed, the ROS was increased in Mn and H_2_O_2_ groups (2.73‐fold and 2.82‐fold, *P* < .01, Figure 7A). Similarly, the ROS was significantly increased in H_2_O_2_ pretreatment group, LV‐PERK shRNA and H_2_O_2_ pretreatment group cells compared with Mn‐treated cells (1.39‐fold and 1.44‐fold, *P* < .01, Figure 7A). To identify the effect of ROS on PERK signalling pathway, then we measured the protein levels of PERK, Phospho‐eIF2α and ATF4, Western blotting results showed that the expression of these proteins were significantly increased in H_2_O_2_ pretreatment group (*P* < .01, Figure 7B, G). Next, we measured the formation of autophagic vacuoles, and H_2_O_2_ pretreatment cells exhibited a significant increase in the formation of autophagic vacuoles compared with Mn‐treated normal cells (1.42‐fold, *P* < .01, Figure 7C). Reversely, in LV‐PERK shRNA group, the formation of autophagic vacuoles was significantly decreased compared with Mn‐treated normal cells (23.63%, *P* < .01, Figure 7C). Then, we measured the expression of beclin 1, LC3II/LC3I and p62, and as the results showed, the expression of beclin 1 and the ratio of LC3II/LC3I protein expression was increased in H_2_O_2_ pretreatment cells compared with Mn‐treated cells (*P* < .01 and *P* < .05). However, the p62 expression was significantly decreased in H_2_O_2_ pretreatment cells compared with Mn‐treated normal cells (*P* < .01, Figure 7D, H). Interestingly, in LV‐PERK shRNA group, the ratio of LC3II/LC3I protein expression was significantly decreased, and the p62 expression was significantly increased compared with Mn‐treated normal cells (*P* < .05, Figure 7D, H). These results indicate that ROS is involved in Mn‐induced autophagy activation, and the PERK signalling pathway plays a very important role in the activation of autophagy.

To validate whether the binding of ATF4 protein to the LC3 promoter is affected by ROS or PERK signalling pathway, we employed ChIP assay and detected the mRNA expression of LC3. As the results showed, the binding of ATF4 to the LC3 promoter was enhanced in H_2_O_2_ pretreatment cells compared with Mn‐treated normal cells (1.43‐fold, *P* < .05, Figure 7E). However, binding of ATF4 to the LC3 promoter and the mRNA expression of LC3 were significantly weakened in LV‐PERK shRNA cells compared with Mn‐treated normal cells (32.95% and 36.39%, respectively, *P* < .05, Figure 7E and F). This suggests that the interaction of ATF4 to the LC3 promoter is directly regulated by the PERK signalling pathway, rather than Mn or Mn‐induced ROS.

## 4 DISCUSSION

Although several studies have explained that ER stress and ER stress–induced apoptosis are concerned with Mn‐mediated neurotoxicity in vivo and vitro,^5^, ^7^ far too little data exist concerning the PERK signalling pathway involved in Mn‐induced protective autophagy, even though this information is vital for better understanding Mn‐induced neurotoxicity. In the current study, we employed cultured SH‐SY5Y cells to evaluate the regulatory function and also the molecular mechanisms of the PERK signalling pathway on the initiation of autophagic. Our findings provide evidence that Mn can decrease cell membrane integrity and viability, thus inducing cell apoptosis.

There are three main apoptosis pathways: the death receptor pathway, the mitochondrial pathway and the ER pathway.^20^ It has been confirmed that Mn can induce apoptosis via the involvement of ER stress and mitochondrial dysfunction.^7^, ^21^ The GRP78 protein is a key regulator in ER stress signalling pathways that are also essential for UPR survival, cell fate and apoptosis responses. However, to cope with excessive ER stress, this protective signalling pathway was changed to a pro‐apoptotic response. The CHOP, also known as growth arrest and DNA damage 153 (GADD153), is a major element of ER stress–mediated apoptosis that strongly depends on ATF4. The CHOP‐mediated pathway is the key pathway in ER stress–mediated cell death.^22^ Furthermore, opposed to other cell death mechanisms, the activation of Caspase‐12 was suggested to be specific to death signals during ER stress and crucial for ER stress–induced apoptosis.^12^ During stress, GADD34 is triggered by ATF4 and CHOP (downstream targets of the PERK signalling pathway) and is participated in the switch between survival and death by dephosphorylating eIF2α.^23^ Additionally, activation of cleavage of some key proteins is considered responsible for cell apoptosis. PARP can be cleaved into 113‐kD and 25‐kD fragments, and the 25‐kD PARP fragment can block DNA repair, thus resulting in cell death. In answering to ER stress, the expression of 25‐kD PARP has been considered a putative molecular marker for apoptosis.^24^ In the current study, our data showed that Mn distinctly increased apoptotic rates. Moreover, there was a significant increase in ER stress–mediated apoptosis markers (GRP78, CHOP, GADD34, cleaved Caspase‐12 and cleaved PARP) in Mn treatment SH‐SY5Y cells. Furthermore, we employed the inhibitor of ER stress, 4‐PBA, as a negative control to study ER stress–mediated apoptosis. We found that ER stress–mediated apoptosis markers were down‐regulated in 4‐PBA‐pretreated cells. Additionally, flow cytometry analysis indicated that apoptosis decreased when ER stress was inhibited. Thus, these investigations support a model in which Mn can activate ER stress and ER stress–mediated apoptosis.

Autophagy is a lysosomal degradation mechanism that maintains homeostasis and represents a response to stress stimuli, including nutrient/energy stress, redox changes, ER stress and mitochondrial damage.^11^, ^25^ To adapt to these conditions, autophagy is initiated, and then, a double‐membrane structure identified as an autophagosome is formed, which then fused with lysosomes to form an autophagic lysosomes, and plays a role in cell death.^26^ However, it is still unclear what role autophagy activation plays in Mn‐induced neurotoxicity. In our study, we found that both autophagy flux and the formation of autophagic vacuoles significantly increased with time and that beclin 1 levels and LC3II/LC3I increased, while p62 expression decreased. These results indicate that Mn can induce autophagy activation. To further examine the effect of autophagy in Mn‐mediated cell apoptosis, we used 3‐MA to inhibit autophagy initiation and found that apoptosis significantly increased in 3‐MA‐pretreated cells. These results suggest that Mn can initiate autophagy activation and play a protective role during cell injury.

The PERK/eIF2α/ATF4 signalling pathway during ER stress regulates the interaction between apoptosis and autophagy in many cell lines, via the effect of ATF4. ATF4 regulates the expression of both LC3 and CHOP/GADD153 by binding to the promoters of LC3B and CHOP/GADD153 in vitro.^18^ Although it has been reported that the PERK signalling pathway is involved in autophagy activation,^27^ direct evidence of the interaction between the of PERK signalling pathway and this upstream regulator of autophagy in Mn‐induced neurotoxicity is still lacking. Our study now demonstrates that the activation of LC3 was regulated by the PERK/eIF2α/ATF4 signalling pathway to protect SH‐SY5Y cells from Mn‐mediated cell injury. These findings imply that ATF4 participates in the regulation of LC3 transcription in answering to Mn treatment. Additionally, after silencing the PERK signalling pathway, we found that Mn‐mediated protective autophagy was inhibited and cell injury was aggravated.

Collectively, our findings demonstrate that initiation of the PERK signalling pathway can participate in the regulation of LC3 expression, which may be responsible for the initiation of autophagy in response to Mn. This not only explains the activation of autophagy in answering to multiple stresses stimuli including ER stress but also provides a progressive mechanism for the regulation of LC3 expression during autophagy.

## CONFLICT OF INTEREST

The authors declare that they have no competing interests.

## AUTHOR'S CONTRIBUTIONS

Chang Liu, Dong‐Ying Yan, Can Wang and Zhuo Ma carried out the experiments and data analyses; Chang Liu, Dong‐Ying Yan and Wei Liu prepared the figures; Bin Xu designed the study and supervised the data; and Chang Liu wrote the manuscript.

## Data Availability

The data used and analysed during the study are available from the corresponding author upon reasonable request.
